# Efficient implementations of a Born Series for computing photoacoustic field from a collection of erythrocytes

**DOI:** 10.1016/j.pacs.2025.100724

**Published:** 2025-04-10

**Authors:** Ujjal Mandal, Navroop Singh, Kartikay Singh, Vinit Nana Hagone, Jagpreet Singh, Anshu S. Anand, Ben T. Cox, Ratan K. Saha

**Affiliations:** a Department of Applied Sciences, Indian Institute of Information Technology Allahabad, Jhalwa, Prayagraj, 211015, Uttar Pradesh, India; b Department of Computer Science and Engineering, Indian Institute of Technology Ropar, Street 29, Ropar, 140001, Punjab, India; c Department of Information Technology, Indian Institute of Information Technology Allahabad, Jhalwa, Prayagraj, 211015, Uttar Pradesh, India; d Department of Medical Physics and Biomedical Engineering, University College London, Gower Street, London, WC1E 6BT, UK

**Keywords:** Helmholtz equation, Green’s function, Convergent Born series, Multiple scattering, Multi-thread/parallel computation, GPU computation

## Abstract

Numerical implementation of the Born series procedure is a computationally expensive task. Various computational strategies have been adopted and tested in this work for fast execution of the convergent Born series (CBS) algorithm for solving inhomogeneous Helmholtz equation in the context of biomedical photoacoustics (PAs). The PA field estimated by the CBS method for a solid circular disk approximating a red blood cell exhibits excellent agreement with the analytical result. It is observed that PA pressure map for a collection of red blood cells (mimicking blood) retains the signature of multiple scattering of acoustic waves by the acoustically inhomogeneous PA sources. The developed numerical tool realizing the CBS algorithm compatible with systems having multiple graphics processing units can be utilized further for accurate and fast estimation of the PA field for large tissue media.

## 1 Introduction

The time-independent wave equation or the Helmholtz equation with a source term arises in many fields of science and engineering. At one hand, it can be applied to estimate seismic wavefield in a highly scattering medium; on the other hand, the phenomenon of electron scattering can also be modeled using this equation [1], [2]. Analytical solutions of inhomogeneous Helmholtz equation can only be obtained for regular scatterers e.g., homogeneous sphere, infinite cylinder etc. For irregular shapes, solutions are evaluated numerically. The most simple numerical approaches include finite difference, finite element methods. Some advanced numerical methods have also been tried. However, solving an inhomogeneous Helmholtz for a large system is challenging.

The Born series method can be explored to solve an inhomogeneous Helmholtz equation as well [3]. It is an iterative approach and therefore, it is a computationally intensive technique [3]. The traditional Born series (TBS) method can provide converging solutions for small particles and small scattering potential problems, but it fails to converge if particle size and scattering potential are large [3]. To tackle this issue, Osnabrugge et al. developed a method which is called the convergent Born series (CBS) [3]. It has been proved that CBS offers converging solutions for the inhomogeneous Helmholtz equation for arbitrarily large contrast. The CBS technique has been successfully implemented to solve inhomogeneous Maxwell’s equations in optical scattering problems [4], [5]. This procedure has also been adopted in the context of biomedical photoacoustics (PAs) to solve the Helmholtz equation with source terms [6], [7], [8], [9]. Huang et al. applied renormalized Born series, also termed as the CBS, for seismic wavefield modeling in strongly scattering media [10]. Recently, Stanziola et al. has reported that machine/deep learning can be utilized to solve the wave equation and referred to as the learned Born series (LBS). It has significantly higher accuracy compared to the CBS protocol for the same number of iterations, especially in the presence of high contrast scatters, while maintaining a comparable computational complexity [11].

Blood is an excellent medium that exhibits the PA effect. PA signals from single-cell level as well as from bulk media have been detected. For instance, Galanza et al. employed diagnostic ultrasound transducers (ranging from approximately 3.5 to 20 MHz) to capture PA signals from various diseased cells, including malaria-infected red blood cells (RBCs), sickle cells, and circulating tumor cells, within living organisms [12], [13], [14]. Strohm et al. utilized ultra-high-frequency transducers, ranging from several hundred to thousand megahertz, to detect PA signals from normal and deformed RBCs [15], [16], [17]. PA spectra for human erythrocytes, stomatocytes and echinocytes have been theoretically estimated as well [18], [19], [20]. Deep vein thrombosis, effect of RBC aggregation on blood oxygenation have also been examined [21], [22]. Bench and Cox (2021) investigated the use of linear unmixing for quantitative PA estimation of intervascular blood oxygenation differences, highlighting its potential for accurate oxygenation mapping [23]. PA assessment of various blood parameters has also been reported [24], [25], [26], [27], [28]. A summary of PA studies aiming to characterize blood pathologies can be found in [29].

The objective of this paper is two fold. First, to develop a computational approach for fast execution of the CBS method. Second, to determine PA field distribution (utilizing such a numerical framework) inside a tissue sample consisting of densely packed acoustically inhomogeneous sources causing multiple scattering of acoustic waves. The numerical implementations were validated by comparing the CBS and analytical results generated by a solid circular disk mimicking a RBC. A tissue sample including non-overlapping RBCs and resembling a blood smear was constructed using the Metropolis–Hastings algorithm [30], [31]. PA pressure distribution within the computational domain was evaluated for a tissue sample by the CBS method and also compared with the analytical results. It is called the discrete particle approach (DPA) in the remaining text. In this procedure, PA fields from individual cells are linearly summed up to obtain the resultant field but it does not take into account the multiple scattering of acoustic waves by inhomogeneous cells. As expected, numerical codes running in a computer having several graphics processing units (GPUs) provided maximum time benefit and outperformed conventional central processing unit (CPU) programming approaches incorporating multi-threading/parallelization. For example, single GPU code found to be approximately 24 times faster than the parallel CPU code. Single GPU execution time scales well with increase in number of GPU resources. PA pressure map for a tissue containing acoustically inhomogeneous cells, provided by the CBS technique, differs from that of the DPA result at all frequencies. It implies that multiple scattering of acoustic waves takes place when non-zero sound-speed contrast for cells with respect to the extra-cellular matrix exists. As far as we know, no work has been done so far investigating this issue in the context of biomedical PAs. This is one of the important contributions of the current work. The numerical approach presented in this study may have applications for accurate determination of the spatial distribution of PA pressure for real tissue.

The organization of the paper is as follows. The governing equations and various approaches for solving such equations are detailed in Section 2. The simulation strategies are illustrated in Section 3. The simulation results and discussion of results are presented in Sections 4, 5, respectively. The conclusions of this study are summarized in Section 6. Different computational platforms utilized in this study are briefly described in Appendix A, Appendix B.

## 2 Theoretical approach

### 2.1 PA wave equation

The time-independent PA wave equation for an acoustically inhomogeneous source is given by [32],

(1a) $$\nabla^{2}\psi \left(r\right)+k_{s}^{2}\psi \left(r\right)=\frac{i\omega \mu \beta I_{0}}{C_{P}},\;\;\;\;\text{within the source}$$

(1b) $$\nabla^{2}\psi \left(r\right)+k_{f}^{2}\psi \left(r\right)=0,\;\;\;\text{in the surrounding medium}$$

where, $k_{s}$ and $k_{f}$ are the wave numbers of the source region and the ambient medium, respectively; the subscripts s and f state the source and the surrounding fluid, respectively; ω indicates the modulation frequency of the exciting light beam with $I_{0}$ being its intensity. Further, μ, β and $C_{P}$ refer to the optical absorption coefficient, isobaric thermal expansion coefficient and specific heat for the absorbing region, respectively. The exact analytical solutions of Eq. (1) can be derived for simple source geometries (e.g., sphere, infinite cylinder, layer, etc.). Briefly, the PA wave equations as described in Eq. (1) are solved in an appropriate coordinate system and thereafter the solutions are matched at the boundary (i.e., continuity of pressure and normal component of particle velocity) [32]. The solution is valid for such an inhomogeneity with arbitrary size and strength. Eq. (1) can also be solved using the approximate approaches, namely, the Born series techniques for regular and irregular shapes. The solution in the case of traditional Born series (TBS) may not always converge [3], [6].

### 2.2 Analytical solutions in 2D

**Fig. 1:**
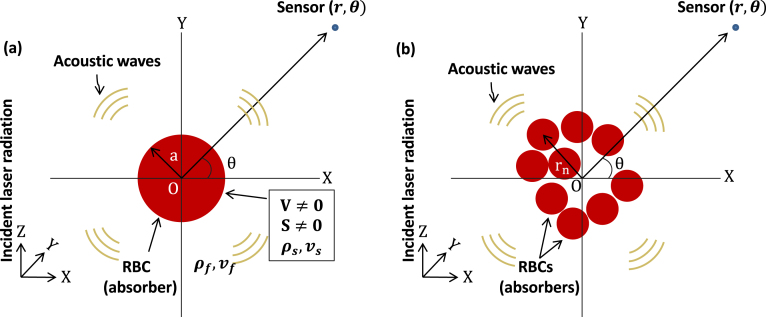
(a) Generation of PA waves from a single-particle system. (b) Emission of PA waves by a many-particle system.

Here we consider a solid circular disk as a PA source. The expressions for the PA field inside and outside the source (a circular solid disk of radius a) becomes [33],

(2a) $$\psi_{s}\left(r\right)=\frac{A}{k_{s}}\left[1+\frac{\overset{ˆ}{\rho }\overset{ˆ}{c}J_{0}\left(k_{s}r\right)H_{1}^{1}\left(k_{f}a\right)}{J_{1}\left(k_{s}a\right)H_{0}^{1}\left(k_{f}a\right)-\overset{ˆ}{\rho }\overset{ˆ}{c}J_{0}\left(k_{s}a\right)H_{1}^{1}\left(k_{f}a\right)}\right],$$

(2b) $$\psi_{f}\left(r\right)=\frac{A}{k_{s}}\left[\frac{J_{1}\left(k_{s}a\right)H_{0}^{1}\left(k_{f}r\right)}{J_{1}\left(k_{s}a\right)H_{0}^{1}\left(k_{f}a\right)-\overset{ˆ}{\rho }\overset{ˆ}{c}J_{0}\left(k_{s}a\right)H_{1}^{1}\left(k_{f}a\right)}\right],$$

respectively. Here, $A=i\mu \beta I_{0}v_{s}/C_{p}$, $\overset{ˆ}{\rho }=\rho_{s}/\rho_{f}$ and $\overset{ˆ}{c}=v_{s}/v_{f}$; ρ and v being the density and speed of sound, respectively. The notations J and $H^{1}$ represent the Bessel function and the Hankel function of first kind, respectively. The subscripts 0 and 1 specify the orders of each function. A representative figure is shown in Fig. 1(a). Eq. (2) is evaluated to calculate the PA field from a single source (referred to as the exact method).

If a collection of light absorbing disks are uniformly illuminated, the corresponding PA field can be cast as,

(3) $$\psi_{DPA}\left(r\right)=A\times \overset{N}{\underset{n=1}{\sum }}\frac{J_{1}\left(k_{s}a_{n}\right)H_{0}^{1}\left(k_{f}\left|r-r_{n}\right|\right)}{k_{s}\left[J_{1}\left(k_{s}a_{n}\right)H_{0}^{1}\left(k_{f}a_{n}\right)-\overset{ˆ}{\rho }\overset{ˆ}{c}J_{0}\left(k_{s}a_{n}\right)H_{1}^{1}\left(k_{f}a_{n}\right)\right]}.$$

Here, the field point is away from the irradiated sources; $r_{n}$ and $a_{n}$ are the position vector and radius of the nth source, respectively. Moreover, the total number of sources considered in this study is N. The resultant PA field is obtained by linearly adding the tiny fields emitted by the individual particles. An illustrative diagram is presented in Fig. 1(b). The light beam propagates along the +ve Z-direction and identically irradiate the cells present in the blood smear. Eq. (3) acts as the mathematical framewrok for the DPA and has been calculated in this work for many-particle systems.

### 2.3 Born series solutions in 2D

The time independent PA wave equation as presented in Eq. (1), after some simple steps, can be rewritten as [3], [6],

(4) $$\nabla^{2}\psi \left(r\right)+\left(k_{f}^{2}+i\epsilon \right)\psi \left(r\right)=-S\left(r\right)-V\left(r\right)\psi \left(r\right),$$

where ϵ is an infinitesimally small real number. The terms on the right hand side are given by,

(5) $$S\left(r\right)=\left\{\begin{array}{cc} -\frac{i\mu \beta I_{0}\omega }{C_{p}}, & \text{if}\left|r\right|\leq a\\ 0, & \text{if}\left|r\right|>a \end{array}\right)$$

and,

(6) $$V\left(r\right)=\left\{\begin{array}{cc} k_{s}^{2}-k_{f}^{2}-i\epsilon , & \text{if}\left|r\right|\leq a\\ -i\epsilon , & \text{if}\left|r\right|>a \end{array}\right)$$

with S(r) and V(r) are the source term and the scattering potential, respectively. It may be pointed out here that ϵ on the left hand side of Eq. (4) makes the medium lossy and thus the medium would attenuate the propagating wave. However, the same term has been added to the scattering potential causing the solution to grow with iteration. These two factors indeed balance each other and facilitate a converging solution.

If the illuminated region contains several identical sources, one can write,

(7) $$S\left(r\right)=\left\{\begin{array}{cc} -\frac{i\mu \beta I_{0}\omega }{C_{p}}, & \text{if}\left|r\right|\leq a_{n};\;n\in 1,\ldots ,N\\ 0, & \text{if}\left|r\right|>a_{n} \end{array}\right)$$

and,

(8) $$V\left(r\right)=\left\{\begin{array}{cc} k_{s}^{2}-k_{f}^{2}-i\epsilon , & \text{if}\left|r\right|\leq a_{n};\;n\in 1,\ldots ,N\\ -i\epsilon , & \text{if}\left|r\right|>a_{n}. \end{array}\right)$$

Representative diagrams are shown in Fig. 1 for single and many particle systems, respectively. The standard practice to solve Eq. (4) is to use the Green’s function method [1], [3]. The Green’s function for the Helmholtz equation satisfies,

(9) $$\nabla^{2}g\left(r\left|r_{0}\right)+\left(k_{f}^{2}+i\epsilon \right)g\left(r\right|r_{0}\right)=-\delta \left(r-r_{0}\right).$$

where, δ is the Dirac delta function. The solution to Eq. (4) using the Green’s function method becomes,

(10) $$\psi \left(r\right)=\int g\left(r|r_{0}\right)\left[V\left(r_{0}\right)\psi \left(r_{0}\right)+S\left(r_{0}\right)\right]d^{3}r_{0}.$$

It is not a trivial task to solve Eq. (10) since the unknown, ψ(r), is also present on the right hand side and therefore, iterative approaches are relied on.

Note that the functional form of the Green’s function in 2D in the far field for a lossy unbounded medium can be derived as [1], [3],

(11) $$g\left(r|r_{0}\right)\approx \frac{i}{4}{\left(\frac{2}{\pi \sqrt{k_{f}^{2}+i\epsilon }|r|}\right)}^{\frac{1}{2}}e^{i\left(\sqrt{k_{f}^{2}+i\epsilon }|r-r_{0}|-\frac{\pi }{4}\right)}.$$

The Green’s function decays exponentially with distance for finite ϵ. As a result of that the function becomes localized as well as its total energy remains finite [3]. The expression for the same function in the Fourier domain is,

(12) $$\overset{\sim }{g}\left(p\right)=\frac{1}{\left({\left|p\right|}^{2}-k_{f}^{2}-i\epsilon \right)},$$

where p is the Fourier transformed coordinates.

#### 2.3.1 Traditional Born series

Eq. (10), which involves convolution sums, in terms of matrices reduces to,

(13) ψ=GVψ+GS,

where $G=F^{-1}\overset{\sim }{g}\left(p\right)F$ where F and $F^{-1}$ are the forward and inverse Fourier transform operators, respectively. Eq. (13) can be recursively expanded yielding,

(14) $$\psi_{TBS}=\left[1+GV+GVGV+\cdots \;\right]GS.$$

Eq. (14) is the famous TBS expression and it converges if GV<1 [3]. In other words, the infinite series converges for small objects with weak scattering potentials.

#### 2.3.2 Convergent Born series

In order to ensure convergence of the TBS protocol for a source of arbitrary size and strength, Osnabrugge et al. proceeded in the following manner. Eq. (13) is multiplied by a preconditioner γ facilitating [3],

(15) γψ=γGVψ+γGS.

After some trivial steps, one arrives at,

(16) $$\psi_{CBS}=M\psi_{CBS}+\gamma GS,$$

where M=γGV−γ+1. As in Eq. (14), an infinite series can be derived by recursively expanding Eq. (16) as,

(17) $$\psi_{CBS}=\left[1+M+M^{2}+\cdots \;\right]\gamma GS.$$

The above series converges when M<1. Osnabrugge et al. proved that the above series converges for all structures if the following choices are made, $\gamma =\frac{i}{\epsilon }V\left(r\right)$ and $\epsilon \geq \text{max}\left(\left|k_{s}^{2}-k_{f}^{2}\right|\right)$ [3]. Eq. (17) has been implemented in this study to compute the PA fields generated by different two-dimensional systems as shown in Fig. 1 and those results have been compared with the analytical results.

**Fig. 2:**
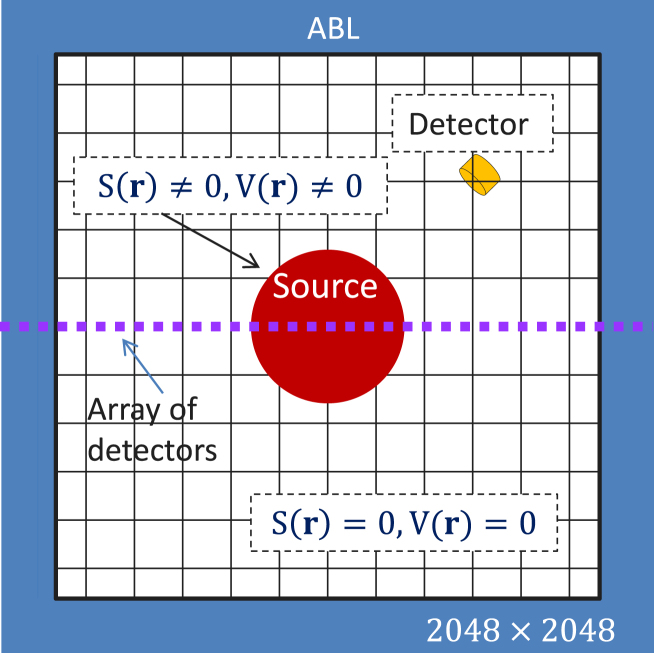
Schematic diagram of the computational domain for implementing the CBS algorithm. ABL signifies the absorbing layer. The lengths/dimensions are not appropriately scaled. Array of point detectors record pressure data along the center line. The PA spectrum is computed at the location of the single detector (marked with yellow) placed outside the source.

## 3 Simulation methods

### 3.1 Single particle system

#### 3.1.1 Calculation of the PA field via the exact method

The PA field from a solid circular disk with radius a=2.75 μm was calculated employing Eq. (2). The disk mimicked a RBC. The density of the source region and the coupling medium was chosen to be, $\rho_{s}=\rho_{f}=1000\;{\mathrm{kg/m}}^{3}$. The speed of sound for the surrounding medium (extracellular matrix) was fixed to $v_{f}=1500\;\mathrm{m/s}$, however, that of the source was decreased from $v_{s}=1950$ to 1200 m/s. In other words, the sound-speed contrast was altered from 0.3 to −0.2. The frequency band for the computation of the PA field was taken to be from 7.3 to 2197 MHz, with an increment of 7.3 MHz (wavelength range became- ≈ 205 to 0.68μm). Accordingly, the size parameter $k_{f}a=\left(2\pi fa\right)/v_{f}$ approximately varied from 0.08 to 25. The numerical values of optical, mechanical, and thermodynamical parameters were set to be unity ($I_{0}$ = 1, μ = 1, β = 1, $C_{p}$ = 1). The PA fields were calculated along the center line at some test frequencies (i.e., 183, 366 and 732 MHz). The PA spectrum was evaluated as well at a distance r=51 μm from the center of the source.

**Fig. 3:**
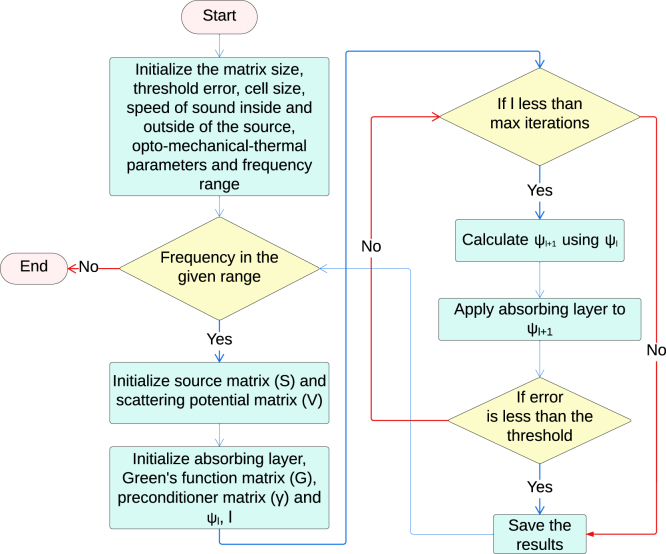
Flowchart describing the steps for implementation of the CBS algorithm.

#### 3.1.2 Estimation of the PA field by the CBS algorithm

A homogeneous circular disk with a diameter of 5.5μm was placed at the center of a square computational domain of size 204.8×204.8 $\;\mu m^{2}$ which was discretized into 2048 × 2048 grid points. The pixel size was $100\times 100\;{\mathrm{nm}}^{2}$. The computational setup is shown in Fig. 2. It is a schematic diagram. The lengths/dimensions are not appropriately scaled. Point detectors were placed to record pressure data along the center line; whereas the PA spectrum was calculated outside the source at the position of the yellow detector. Fig. 3 depicts the workflow of the CBS scheme. Algorithm S1 elaborates the CBS protocol. At first, the spatial maps of S(r) and V(r) were generated by deploying Eqs. (5), (6). The next step was to calculate the Green’s function in the frequency domain, see Eq. (12), for $\epsilon =0.8k_{f}^{2}$ [3]. Accordingly, the acoustic attenuation coefficient (α) of the computational domain could be estimated to be $\alpha \approx \frac{\epsilon }{2k_{f}}$, which provided α=122.8 Np/cm at 7.32 MHz. Note that this quantity for the breast tissue is about 1.71 Np/cm or 14.85 dB/cm at the same frequency.

The initial pressure distribution was computed to be,

(18) $$\psi_{{CBS}_{0}}\left(r\right)=\gamma \left({\text{ifft}}_{2}\left[\overset{\sim }{g}\left(p\right){\text{fft}}_{2}S\left(r\right)\right]\right).$$

The notations fft${}_{2}$ and ifft${}_{2}$ denote the forward and backward fast Fourier transforms (FFTs) in 2D, respectively. Further, all the multiplications were carried out element-wise. Thereafter, iterative steps were performed such as,

(19) $$\psi_{{CBS}_{l+1}}\left(r\right)=\psi_{{CBS}_{l}}\left(r\right)-\frac{i}{\epsilon }V\left(r\right)\left(\psi_{{CBS}_{l}}\left(r\right)-\right)\;\left({\text{ifft}}_{2}\left[\overset{\sim }{g}\left(p\right){\text{fft}}_{2}\left[V\left(r\right)\psi_{{CBS}_{l}}\left(r\right)+S\left(r\right)\right]\right]\right),$$

with l being the iteration number.

It might be mentioned here that an absorbing layer (ABL) was attached at each boundary of the computational domain, see Fig. 2. The PA waves while moving through this layer were greatly attenuated or in other words, the outgoing waves essentially did not reflect back from the boundaries. The sigmoid function was used to model the absorbing layer and it is defined as,

(20) $$ABLFn\left(x\right)=\frac{1}{1+\exp\left(-\kappa \left(x-0.5d\right)\right)},\text{within the ABL}$$

where x>0 and d is the thickness of the ABL (d=500 grid points); κ was fixed to, $\kappa =61.36\times 10^{2}$ Np/cm for all frequencies. The pressure field was multiplied by the window function and accordingly, updated as $\psi_{l+1}=ABLFn\times \psi_{l+1}$. In order to test the convergence of the PA field, the total error for the center line was obtained and it was defined as,

(21) $$\text{Total error}=\frac{\overset{2048}{\underset{m=1}{\sum }}\left|\psi_{l+1}\left(1024,m\right)-\psi_{l}\left(1024,m\right)\right|}{\overset{2048}{\underset{m=1}{\sum }}\left|\psi_{l}\left(1024,m\right)\right|}.$$

It was assumed that the steady state was attained if the total error was less than a threshold value (i.e., 10^−4^). The iterative calculation stopped once the steady state was reached. A maximum 2000 iterative steps were allowed to yield the converging solution. The PA fields were found to be converged within 2000 iterative steps for all frequencies and for the size of the computational domain considered in this study. If the total error was more than the threshold value, the latest PA field was assigned to the previous PA field, i.e., $\psi_{l}=\psi_{l+1}$. The subsequent step was to use $\psi_{l}$ as an input for Eq. (19) and hence a new estimation was made.

It is apparent from Eq. (19) that it includes convolution sums and those were evaluated in the frequency domain. Therefore, the forward and inverse FFTs were computed extensively in CBS method. The FFT inherently applies the periodic boundary condition, which means a wave emerging out from a boundary reappears from the opposite boundary. The ABL greatly discards such a possibility and hence the predicted fields are evaluated for the outgoing waves only. As mentioned earlier, the CBS method converges if $\epsilon \geq \text{max}\left(\left|k_{s}^{2}-k_{f}^{2}\right|\right)$. In this work, we choose, $\epsilon =0.8k_{f}^{2}$ and such a choice always satisfied the above condition even though $v_{s}$ varied from 1200 to 1950 m/s. A MATLAB code realizing the CBS algorithm can be found in [34].

#### 3.1.3 Numerical implementations

In this work, we had to carry out different matrix operations like initialization, addition, multiplication, FFT and IFFT etc. on large matrices (2048 × 2048). The performance could be boosted by using parallelization techniques on these operations. Thus, different optimization techniques were incorporated into the CPU and GPU codes. Appendix A, Appendix B detail the approaches considered in this study. The specifications of the computational resources are given in Table 1.

**Table 1:** Description of the computational resources used in this study.

Feature	Description
CPU model	Intel(R) Xeon(R) Gold 5218R CPU @ 2.10 GHz
Number of CPU cores	40
Cores per CPU	20
Thread(s) per core	1
Core(s) per socket	20
Architecture	x86_64, 64-bit
Operating system (OS)	CentOS Linux 7 (Core)
Storage	251 GB RAM, 2355 GB Disk

GPU model	NVIDIA GeForce RTX 3090
Number of CUDA cores	10496 per GPU
GPU driver version	515.76
CUDA toolkit version	9.2
Number of GPUs	4
GPU RAM	24 GB per GPU

### 3.2 Densely packed many-particle system

#### 3.2.1 Generation of tissue configurations

**Fig. 4:**
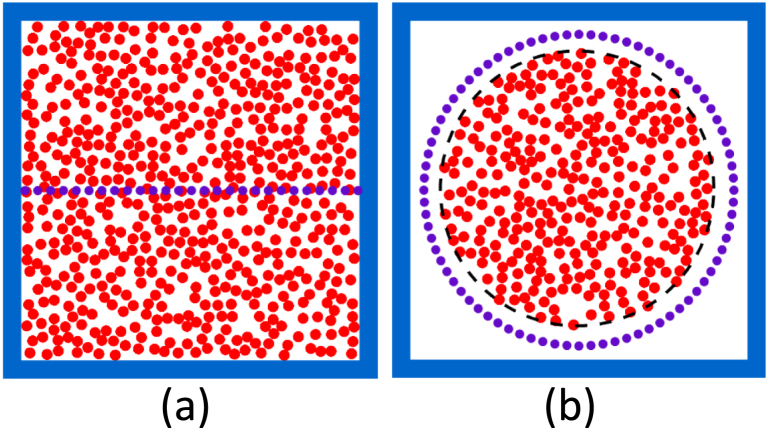
(a) Illustration of a simulated tissue realization. A total 403 solid circular disks (resembling RBCs) are randomly distributed within the ROI (leaving the ABL, blue strips) achieving 40% hematocrit level. The PA pressure data are collected along the center line (filled violet dots). (b) Presentation of another tissue configuration; 133 cells approximated as disks are arranged in a circular region with radius 50μm; a circular array of detectors are placed at a radius of 51μm from the center of the ROI.

The efficacy of the CBS scheme was further tested on a tissue sample, mimicking a blood smear. The solid disks approximating RBCs were randomly placed within the region of interest (ROI) to generate a tissue configuration (leaving the absorbing layers). The size of the ROI was 1048 × 1048 grid points or 104.8×104.8 μm ${}^{2}$. The disks did not overlap in an acceptable tissue realization. The well-known Metropolis–Hastings algorithm was employed for this purpose [30], [31]. The disks were initially randomly placed within the ROI- a cell could be placed anywhere within the ROI (grid crossings as well as any other locations). Then the total energy of the system was calculated by summing up the energies of the overlapping disks ($E=\sum_{i\neq j}V_{ij}$). The interaction energy for a overlapping pair was assigned to be $V_{ij}=1000\;k_{B}T$, where $k_{B}$ is the Boltzmann constant and T is the temperature of the system in the Kelvin scale. After that one particle was picked randomly and thrown into a new position, which was also randomly chosen. The Metropolis–Hastings protocol was then deployed to decide whether the new arrangement had to be accepted or rejected. The energy levels of these states (old and new) were compared in this algorithm. The proposed move was accepted if the energy difference between the states, (ΔE=energy of the new configuration — energy of the old configuration) was negative. Otherwise, the Metropolis ratio ($e^{-\Delta E/k_{B}T}$) was computed and compared with a random number. The move was accepted if the random number was less than or equal to that ratio; the move was rejected if this condition was not fulfilled. For a valid move, the coordinates of the particle were renewed otherwise old coordinates were retained. The Metropolis iterations were continued until the total energy of the system became 0. The steps are summarized in Algorithm S2. The simulated tissue configurations, i.e., ensembles of non-overlapping solid disks, are shown in Fig. 4. The cells occupied a 40% area of the ROI i.e., the square area leaving the ABL in Fig. 4(a) and the circular region bounded by the dashed line in Fig. 4(b). In other words, 403 and 133 cells were placed in Fig. 4(a) and (b), respectively. The PA pressure data were stored for the detector locations (marked by the solid violet dots).

#### 3.2.2 Computation of the PA field

The PA pressure data were calculated for a collection of disks along the center line for the test frequencies. The PA spectra were computed as well at the circularly placed detectors [at a distance r=51 μm from the center of the ROI, see Fig. 4(b)]. Eq. (3) was used for the analytical method. The numerical steps for the CBS method were the same as that of the single particle system. Nevertheless, S(r) and V(r) matrices were built based on the locations of the disks and utilizing Eqs. (7), (8), respectively. Algorithm S3 details the computational steps.

Moreover, variation of magnitude of PA pressure with sound-speed contrast was also examined in this work. To do so, the average PA pressure for the center line, see Fig. 4(a), was calculated at a specific frequency of 183 MHz for a tissue realization and after that the ensemble average of the same quantity was estimated utilizing 100 tissue realizations. The same study was also repeated at 366 and 732 MHz. The GPU CBS code was utilized for these simulations so that results could be obtained within a minimum time.

**Fig. 5:**
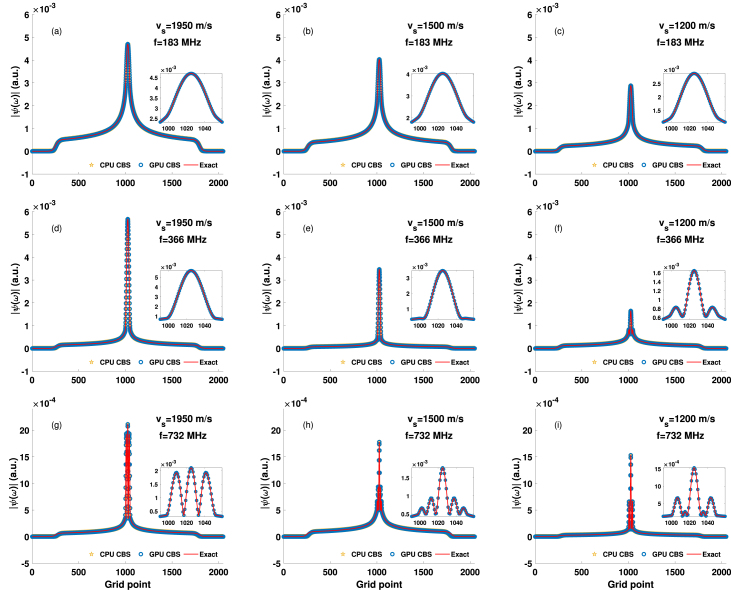
Variation of the PA fields (along the X-axis), generated by the exact and CBS methods, at various frequencies for a circular source with a=2.75μm and when $v_{f}=1500\;\mathrm{m/s}$. (a)–(c) Plots of the PA field calculated at 183 MHz when $v_{s}=1950$, 1500, 1200 m/s, respectively. (d)–(f) and (g)–(i) Same as the top row but for 366 and 732 MHz, respectively.

**Fig. 6:**
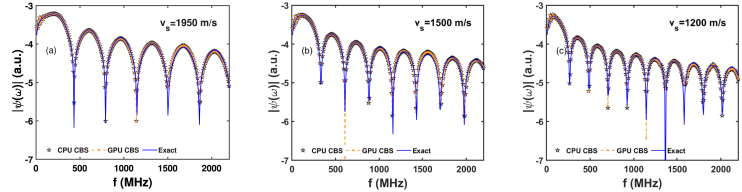
Visualization of the PA spectra simulated for different speed of sound contrasts. The PA field is generated by a circular source with a=2.75μm and the detector is 51μm away from the center of the source. (a)–(c) Speed of sound inside the source ($v_{s}$) decreases gradually from left to right but the same quantity outside the source remains constant ($v_{f}=1500\;\mathrm{m/s}$).

## 4 Computational results

### 4.1 Single particle system

#### 4.1.1 Carrier wave along the center line

All figures in Fig. 5 present how the amplitude of the carrier wave progresses at the steady state along the center line of the computational domain for a solid circular PA source. The numerical values for the speed of sound for the source region are assigned to be $v_{s}=1950$, 1500 and 1200 m/s for the first, second and third columns, respectively. The first, second and third rows contain absolute values of the pressure fields for f = 183, 366 and 732 MHz, respectively. The central part is magnified and shown in the inset to display the oscillations. Approximately 39, 40 and 42 iterations have been required to achieve the steady state, respectively for f = 183 MHz. The results obtained by the CBS protocol (for CPU and GPU implementations) are compared with that of the exact method. The CPU and GPU implementations provide almost the same estimation. It is clear from Fig. 5 that the PA pressure determined by the CBS algorithm exhibits perfect match with the exact approach inside as well as outside of the source. The amplitude of the PA pressure decreases as $v_{s}$ decreases [compare Fig. 5(a), (b) and (c)].

**Fig. 7:**
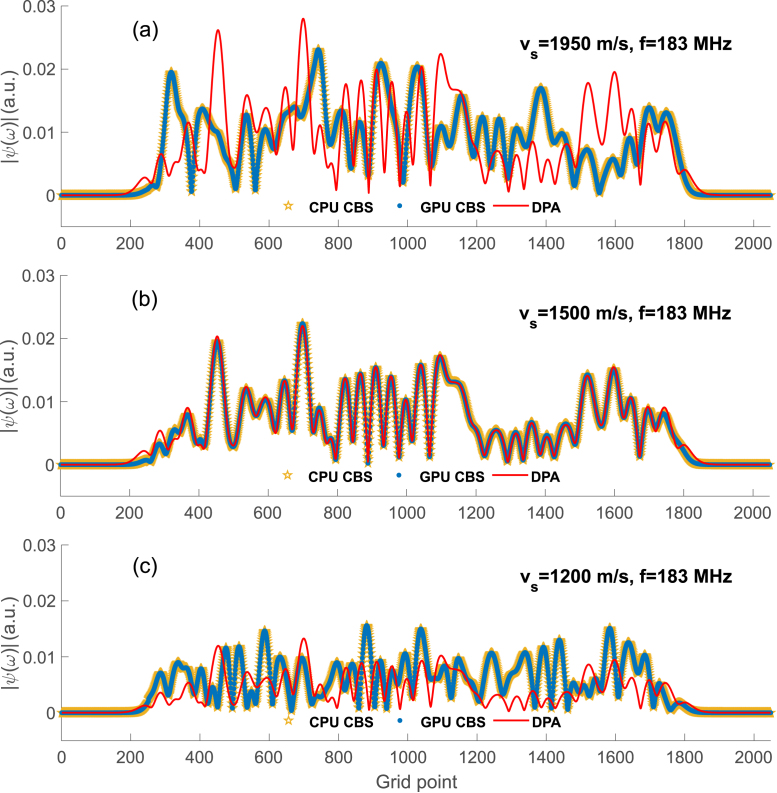
Plots of PA pressure computed at 183 MHz developed by a tissue realization along the center line [see Fig. 4(a)] at different sound-speed contrast conditions; $v_{s}=1950$, 1500, 1200 m/s for (a), (b), (c), respectively.

**Fig. 8:**
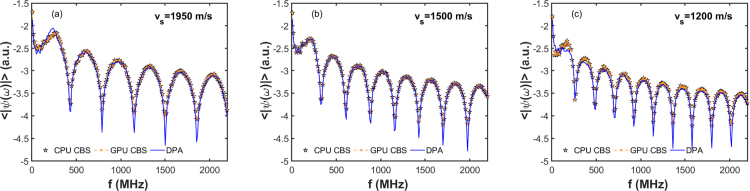
Delineation of the average PA spectrum for a representative tissue configuration predicted by the DPA and CBS methods over a large frequency band (7.32 to 2197 MHz). The ensemble average has been computed for 200 circularly placed detectors [see Fig. 4(b)]. The sound-speed contrast changes from 30% to −20% in (a)–(c), respectively.

#### 4.1.2 Variation of the PA spectrum

The variation of PA field as a function of frequency over a large frequency band (7.32 to 2197 MHz) is shown in Fig. 6. The PA field is generated by a 2D source of radius, a=2.75μm and the field point is located at a distance 51μm from the center of the computational domain. The speed of sound for the source region gradually decreases from left to right in Fig. 6. It is clear from this figure that the number of maxima/minima increases as the speed of sound is decreased from $v_{s}=1950$ to 1200 m/s [see Fig. 6(a), (b) and (c)]. Furthermore, the spacing between two successive minima also decreases as we move from Fig. 6(a) to (c). Note that the first minimum occurs approximately at 432, 330 and 264 MHz for $v_{s}=1950$, 1500 and 1200 m/s, respectively. The CBS simulations demonstrate excellent agreement with the exact method in the entire frequency range.

### 4.2 Densely packed many-particle system

#### 4.2.1 Carrier wave along center line

Representative plots of the magnitude of PA pressure along the X-axis (center line of the computational domain) for a tissue realization at 183 MHz are shown in Fig. 7. The tissue configuration consists of a collection of disks mimicking RBCs, which are randomly distributed within the region of interest attaining 40% hematocrit [see Fig. 4(a)]. The Metropolis–Hastings algorithm has been employed to generate the random locations of the non-overlapping disks. The PA pressure provided by the various theoretical frameworks is a complex quantity and therefore its amplitude is plotted. The sound-speed contrast is positive for the first row (by 30%) and negative for the third row (by 20%). For the second row, it is nil because the PA sources are acoustically homogeneous. Fig. 7 demonstrates that both the CBS implementations produce the identical results. It is interesting to note that the CBS results deviate greatly from the DPA counterparts when acoustic contrast is nonzero [see the first and third rows of Fig. 7]. Similar plots for f = 51, 73, 103, 366 and 732 MHz are shown Figs. S1, S2, S3, S4 and S5, respectively. It seems that the PA pressure inside the tissue sample depends upon the acoustic properties of the cells.

#### 4.2.2 Variation of the average PA spectrum

Typical average PA spectra for the tissue sample considered in this study [see Fig. 4(b)] are presented in Fig. 8. The PA spectra have been calculated at 200 detector locations and accordingly, the average spectrum is obtained. The frequency bandwidth is considered to be 7.32 to 2197 MHz. The simulated spectra for three cases with $v_{s}=1950$, 1500 and 1200 m/s are presented in Fig. 8(a) to (c), respectively. The spectral amplitudes are in general higher in this case than that of Fig. 6. Otherwise, the spectral features of Fig. 8 are analogous to that of Fig. 6. Therefore, the average PA spectrum for a many-particle system essentially reproduces the corresponding single-particle spectrum under this test condition. However, the PA spectrum contains lots of fluctuations if the spectral data recorded by one of the detectors are plotted (data not shown).

## 5 Discussion

The time independent inhomogeneous PA wave equation, Eq. (4), contains two source terms on the right hand side. The first term is responsible for conversion of optical energy into acoustical energy. For an acoustically homogeneous source, the solution of Eq. (4) can be accomplished easily. The second term can be recognized as a scattering potential and it occurs when the speed of sound inside and outside the source is not the same. It acts as a potential well when $v_{s}>v_{f}$ (or $k_{s}<k_{f}$) and as a potential barrier when $v_{s}<v_{f}$ (or $k_{s}>k_{f}$). For an acoustically inhomogeneous source, obtaining the solution of Eq. (4) involving the Green’s function approach is not trivial. The pressure field inside the source needs to be known a priori in order to find a solution. To address this issue, iterative approach has been developed i.e., the TBS scheme. The CBS technique further extends the validity domain of the TBS protocol.

**Fig. 9:**
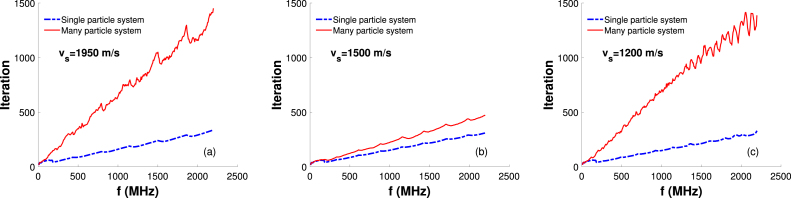
Plots of iteration required for the CBS method to converge versus frequency for single-particle and many-particle systems. (a)–(c) Speed of sound inside the source is considered to be $v_{s}=1950$, 1500 and 1200 m/s, respectively.

Osnabrugge et al. proved that the CBS method converges if γ and ϵ are suitably chosen. In this work, we found that PA field calculations for all cases converged within 337 iterations when the computational domain included a single acoustically inhomogeneous source and the same number became 1451 for the many-particle systems. Fig. 9 displays (blue dashed and red lines) how many iterations were required for convergence at various probing frequencies for the single-particle and many-particle systems. The difference between the blue and red lines becomes prominent as the magnitude of sound-speed contrast as well as the frequency are increased. For example, it can be calculated from Fig. 9(a) for $v_{s}=1950\;\mathrm{m/s}$ that 42 and 148 iterations are required for convergence at f = 183 MHz for the single-particle and many-particle systems, respectively; whereas these values become 284 and 1204, respectively at f = 1831 MHz. A similar pattern can also be seen from Fig. 9(c), i.e. for $v_{s}=1200\;\mathrm{m/s}$. The difference is negligible when the sound-speed contrast is zero, see Fig. 9(b). Therefore, it is observed that the CBS method takes more steps to converge for the many-particle system than that of the corresponding single-particle system.

**Fig. 10:**
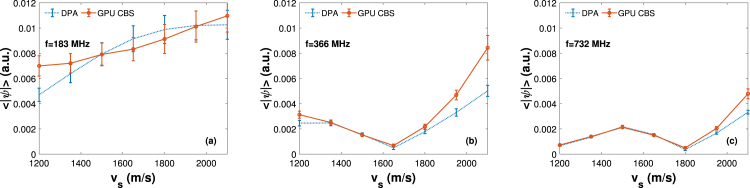
Magnitude of mean PA field (± standard deviation) by various speed of sound mismatch at f = 183, 366 and 732 MHz.

One of the important aspects of the Born series method is that S(r) and V(r) matrices can accommodate multiple sources of arbitrary shapes and strengths. Note that an acoustically inhomogeneous PA source would act as a scatterer for waves generated by the other sources. The Born series framework implicitly incorporates such interactions. It starts with initial pressure fields assigned to the individual sources but those fields interact with each other and spread as the iteration progresses. And finally, steady state condition is reached. Therefore, it holds the possibility of multiple scattering of acoustic waves. This issue has also been investigated in this work. We computed PA pressure data on the grid points along the center line for a tissue sample at a particular frequency and subsequently, mean value was obtained. The same simulation was repeated for 100 tissue realizations and accordingly, ensemble average (± standard deviation) was obtained. Fig. 10(a)–(c) exhibit how the ensemble average of PA pressure inside the tissue varies with increasing sound-speed mismatch at f =183, 366 and 732 MHz, respectively. The same quantity predicted by the DPA is also shown in each figure for comparison. It is clear from Fig. 10 that the exact and CBS results do not agree when the sound-speed contrast is large. Therefore, multiple scattering of acoustic waves might have played a role and that is why the PA pressure developed inside the tissue and predicted by the CBS technique does not agree with the same quantity estimated by the DPA. In contrary, no observable effect of multiple scattering is seen outside the tissue region (compare the PA spectra of CBS and DPA methods in Fig. 8). Therefore, modification of pressure field due to multiple scattering of acoustic waves by acoustically inhomogeneous PA sources can be estimated using the CBS method. It is anticipated that the findings of this study may be useful for PA microscopy (for improved tissue profiling). However, further investigations are required to achieve this end involving a real detector sensing PA signals at realistic distances.

**Fig. 11:**
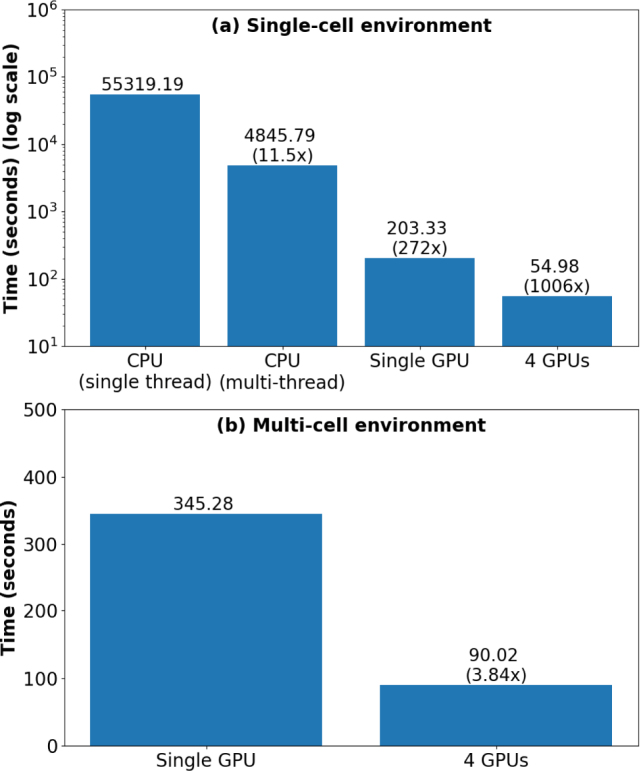
Time taken to compute PA fields for 300 frequencies with $v_{s}=1500$ and $v_{f}=1500$ for (a) single-cell environment, (b) multi-cell environment.

Note that it is a proof of the concept work numerically examining the effect of multiple scattering of acoustic waves by acoustically inhomogeneous cells as mentioned above. Therefore, a large bandwidth has been considered from 7.3 to 2197 MHz in order to show the robustness of the iterative framework. It may be emphasized here that acoustic damping grows non-linearly with frequency posing a challenge to detect high frequency acoustic signals in practice. PA spectra in this work were calculated at r=51 μm from the center of the computational domain due to the restriction imposed by the size of the computational domain (2048 × 2048). A larger computational domain (e.g., 4096 × 4096), could allow r to be increased further. Experimental (PA and acoustic microscopy) studies preferred to place the sample at the focal region to maximize signal-to-noise ratio [35], [36], [37], [38].

In this work, cells have been assumed to be uniformly illuminated by the incident optical beam and accordingly, Eq. (3) has been computed. This equation has to be ameliorated if the cells are not identically irradiated (in that case $A=A_{n}=i\mu \beta I_{0n}v_{s}/C_{p}$ is to be placed inside the summation, $I_{0n}$ is the incident light intensity for the nth cell). Analogously, S matrix would be updated accounting each source before implementation of the CBS algorithm. It will be interesting to study this aspect in future and subsequently, compare the CBS and DPA results.

Fig. 11 depicts the total time taken to obtain the solution through the CBS method using CPU and GPU with different parallelization techniques. As one can see that, in case of single cell environment, the single threaded CPU code took approximately 5.37 h. Using parallelization in CPU, the execution time was reduced to approximately 1.35 h. By moving the operation from CPU (multi-thread) to single GPU, about 24x gain in the performance could be achieved, reducing the time to mere 3.4 min. Further reduction of execution time was accomplished by utilizing all the GPUs present in the system by which almost 4x speed-up in performance was attained (using 4 GPU setup). Overall, the execution time was reduced from 15.37 h to just 55 s, which is 1006x faster. In multi-cell environment, the single GPU took 5.75 min and 4 GPUs took 1.5 min (which is about 4x faster).

The ABL, in this work, has been taken to be 500 grid points. The sigmoid function very slowly decreases from 1, which ensures no reflection from the boundaries and also attains a very small value at each outermost edge warranting that the wave would not reappear (wrap around) from the opposite boundary. Accordingly, the simulation results demonstrate that this ABL provides reliable estimations of the PA fields at all frequencies. Nevertheless, the width of the ABL considered herein is thicker than expected and therefore, reduces the size of the ROI which contains the RBCs. The thickness of the absorbing layer typically used in k-Wave simulations is also pretty thin (10 to 20 grid points) [39]. Recently, a new method has been developed which works with ultra-thin ABL [40]. In future, we would like to explore various approaches to reduce the thickness of the ABL, increasing the ROI. Moreover, this numerical framework may also be used for PA field calculation by 3D systems.

## 6 Conclusions

In conclusion, a computational tool for fast realization of the CBS protocol has been developed to calculate spatially varying PA pressure data originating from a single RBC or many RBCs. Tremendous computational speed-up has been achieved by running the codes in a machine with multiple GPUs compared to other typical architectures. Almost 90 times speed-up has been gained i.e., multi-GPU (4 GPUs) code with respect to the CPU code implementing multi-threading. For the single particle system, the CBS method perfectly reproduces the analytical result. In case of a tissue medium, it is intuitively expected that acoustic waves generated by individual PA sources would interact with other acoustically inhomogeneous sources and thus modify the pressure field inside the tissue. This simulation framework provides a means to study this aspect and it may find many applications in future.

## CRediT authorship contribution statement

**Ujjal Mandal:** Writing – review & editing, Writing – original draft, Visualization, Validation, Methodology, Formal analysis, Data curation, Conceptualization. **Navroop Singh:** Writing – review & editing, Writing – original draft, Visualization, Validation, Formal analysis, Data curation. **Kartikay Singh:** Writing – review & editing, Writing – original draft, Visualization, Validation, Methodology, Formal analysis, Data curation. **Vinit Nana Hagone:** Writing – review & editing, Writing – original draft, Visualization, Validation, Methodology, Formal analysis, Data curation, Conceptualization. **Jagpreet Singh:** Writing – review & editing, Writing – original draft, Visualization, Validation, Methodology, Formal analysis, Data curation, Conceptualization. **Anshu S. Anand:** Writing – review & editing, Writing – original draft, Visualization, Validation, Methodology, Data curation, Conceptualization. **Ben T. Cox:** Writing – review & editing, Writing – original draft, Visualization, Validation, Supervision, Investigation, Data curation. **Ratan K. Saha:** Writing – review & editing, Writing – original draft, Visualization, Validation, Supervision, Methodology, Funding acquisition, Formal analysis, Data curation, Conceptualization.

## Declaration of competing interest

The authors declare that there are no competing interests.

## Figures and Tables

**Fig. 12:**
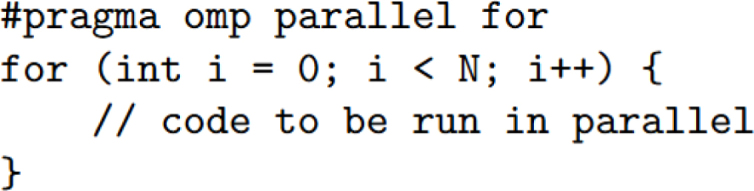
Usage of OpenMP for parallelization of a for loop.

## Data Availability

Data will be made available on request.

## Appendix A CPU implementation

This section discusses the implementation of the CBS method using CPU only. A C++ code was developed to implement the CBS algorithm; C++ language is known for its efficiency and flexibility. To improve the execution time, following parallelizations were done.

**OpenMP:** OpenMP (Open Multi-Processing) is an application programming interface (API) that supports multi-platform shared memory multiprocessing programming in C and C++ [41]. It provides a simple and flexible interface for developing parallel applications on platforms from the desktop to the supercomputer. OpenMP is used in this implementation to parallelize loops, which can significantly improve performance on multi-core processors. The #pragma omp parallel for directive tells the compiler to distribute the loop iterations across the available threads. Each thread will execute a portion of the loop independently of the others. Fig. 12 shows the corresponding syntax.

**FFTW library:** The FFT and inverse FFT are needed to be computed for different matrices in every iteration. The FFT and IFFT are computationally intensive tasks. We can reduce the computational time by using multithreading. FFTW library is one such implementation that uses multithreading and various other optimization techniques to compute FFTs faster.

## Appendix B GPU implementation

This work involved operations with large matrices and therefore, we could have even faster computation using GPU. This section details how the CBS algorithm was realized in GPU. The Compute Unified Device Architecture (CUDA) was employed to parallelize matrix computations using GPU [42].

CUDA is a parallel computing platform and API. It leverages the processing power of GPUs for general-purpose applications beyond traditional graphics processing. This approach, known as General-Purpose computing on GPUs (GPGPU), utilizes CUDA as a software layer to provide direct access to the GPU’s architecture and its parallel processing capabilities. This facilitates the execution of specialized code blocks, termed compute kernels, on the GPU for significant performance gains in computationally intensive tasks.

The memory requirement of our program was about 500−600 MB. All the matrices were directly allocated in the GPU memory. This step reduced the overhead posed by the copy operation from host to device. The iterations for the for-loops from lines 8 to 21 in Algorithm S1 are independent of each other. Hence, these loops were parallelized using a GPU kernel in which each GPU thread computed lines 10 to 21 for every index. GPU kernels were also implemented to perform different matrix operations like addition, subtraction, multiplication (see line 24 in Algorithm S1) etc. CuFFT was used to compute FFTs and inverse FFTs in GPU [43]. It might be mentioned here that launching a CUDA kernel involves some overhead for setting up the execution environment and therefore, special care was given to accomplish various tasks with a minimum number of kernels, leading to performance gains.

The execution speed can be further enhanced in systems where multiple GPUs are available. In the present work, the frequency loop was divided into four sets and each set was executed in each GPU system. For example, GPU 1 did the computations for frequencies {1, 5, 9, …}, GPU 2 was engaged for frequencies {2, 6, 10, …} and so on. This approach became the fastest among all.

In many-particle system, the S and V matrices had to be updated for every cell. In order to do so, we built a cell_mask (see lines from 2 to 14 in Algorithm S3). The points of this matrix within the cells were marked first. Using this cell_mask, S and V matrices were initialized (as depicted in lines from 12 to 19 of Algorithm S3). This scheme initialized S and V matrices once rather than modifying these matrices for every cell leading to improved performance.

## Appendix C Supplementary data

MMC S1 Photoacoustic field calculation from blood using a Born series method.
