# Supplementary material for: Efficient implementations of a Born Series for computing photoacoustic field from a collection of erythrocytes
Source: Photoacoustics. 2025 Apr 10;43:100724. doi: 10.1016/j.pacs.2025.100724 (PMC12019201; doi:10.1016/j.pacs.2025.100724)
Supplement: MMC S1 — Photoacoustic field calculation from blood using a Born series method. [file mmc1.pdf]

Supplementary materials

Algorithm S1: PA field calculation using the CBS algorithm.

Input : Initialize system parameters:
Prepare the computational domain: N_{cn}, N_{cn}, K_{cn}, dx, dy, ABL
Convergence limit: $ThError$
Properties of the source: a, v_s, μ, β, C_P
Properties of the medium: v_f
Intensity of light beam: I_0
Number of frequencies: N_F
Output: PA field ψ_{fn}

for $i = 1$ **to** N_F **do**
  $f \leftarrow i \times v_f/(N_{cn} \times dx)$
  $\omega \leftarrow 2 \times \pi \times f$
  $k_s \leftarrow \omega/v_s$
  $k_f \leftarrow \omega/v_f$
  $\epsilon \leftarrow 0.8 \times k_f^2$
  $\alpha \leftarrow 100 \times 0.5 \times 0.8 \times k_f$
  for $j = 0$ **to** $N_{cn} - 1$ **do**
    for $m = 0$ **to** $N_{cn} - 1$ **do**
      $index \leftarrow j \times N_{cn} + m$
      $dist \leftarrow \sqrt{(m - K_{cn})^2 + (j - K_{cn})^2} \times dx$
      if $dist \leq a$ **then**
        $S[index] \leftarrow (0.0, -(\mu \times \beta \times I_0 \times \omega)/C_p)$
        $V[index] \leftarrow (k_s^2 - k_f^2, -\epsilon)$
      else
        $S[index] \leftarrow (0.0, 0.0)$
        $V[index] \leftarrow (0.0, -\epsilon)$
      $\gamma[index] \leftarrow \imath \times V[index]/\epsilon$
      $k_x \leftarrow 2\pi(m - K_{cn})/(N_{cn} \times dx)$
      $k_y \leftarrow 2\pi(j - K_{cn})/(N_{cn} \times dy)$
      $G[index] \leftarrow \left(\frac{k_x^2+k_y^2-k_f^2}{(k_x^2+k_y^2-k_f^2)^2+\epsilon^2}, \frac{\epsilon}{(k_x^2+k_y^2-k_f^2)^2+\epsilon^2}\right)$
  $G \leftarrow fftShift(G)$
  $\psi_{in} \leftarrow \gamma \times ifft(G \times fft(S))$
  for $iter = 1$ **to** 2000 **do**
    $\psi_{fn} \leftarrow (\psi_{in} - (\imath/\epsilon) \times V \times (\psi_{in} - ifft(G \times fft(V \times \psi_{in} + S)))) \times ABLFn$
    $error \leftarrow \frac{\text{norm}(\text{abs}(\psi_{fn}[N_{cn}/2,:] - \psi_{in}[N_{cn}/2,:]))}{\text{norm}(\text{abs}(\psi_{in}[N_{cn}/2,:]))}$
    if $error < ThError$ **then**
      $saturationCBS \leftarrow iter$
      break
    else
      $\psi_{in} \leftarrow \psi_{fn}$

Algorithm S2: The Metropolis-Hastings algorithm for construction of a tissue configuration.

Input : Initialize system parameters:
  Set the ROI: L_x, L_y
  PA source parameters: a, N, x_{old}, y_{old}
  Interaction energy: $V = 1000k_BT$
Output: Final configuration: x_{new}, y_{new}
Compute initial energy: $E_{\text{old}} = \sum_{i \neq j} V_{ij}$
for $kk = 1$ **to** 500000 **do**
  Propose location of a disc: x_{new}, y_{new}
  Calculate new energy: $E_{\text{new}} = \sum_{i \neq j} V_{ij}$
  Determine: $\Delta E = E_{\text{new}} - E_{\text{old}}$
  if $\Delta E \leq 0$ **then**
    Accept the new configuration
    $\mathbf{r}_{\text{old}} \leftarrow \mathbf{r}_{\text{new}}$
    $E_{\text{old}} \leftarrow E_{\text{new}}$
  else
    Rn = random number
    if $\exp(-\Delta E/k_BT) \geq Rn$ **then**
      Accept the new configuration
      $\mathbf{r}_{\text{old}} \leftarrow \mathbf{r}_{\text{new}}$
      $E_{\text{old}} \leftarrow E_{\text{new}}$
    else
      Reject the new configuration
      $\mathbf{r}_{\text{new}} \leftarrow \mathbf{r}_{\text{old}}$
      $E_{\text{new}} \leftarrow E_{\text{old}}$
  if $E_{new} < 0.001$ **then**
    Break

Algorithm S3: Modification in Algorithm S1 for many-particle system

Input : Same as Algorithm S1
for *each cell in cell_positions* **do**
  for $j = 0$ **to** $N_{cn} - 1$ **do**
    for $m = 0$ **to** $N_{cn} - 1$ **do**
      $index \leftarrow j \times N_{cn} + m$
      $dist \leftarrow \sqrt{(j - \text{cell.y})^2 + (m - \text{cell.x})^2} \times dx$
      if $dist \leq a$ **then**
        $cell\_mask[j][m] \leftarrow$ true
      else
        $cell\_mask[j][m] \leftarrow$ false
for $i = 1$ **to** N_F **do**
  // Code
  for $j = 0$ **to** $N_{cn} - 1$ **do**
    for $m = 0$ **to** $N_{cn} - 1$ **do**
      if $cell\_mask[j][m] = true$ **then**
        $S[index] \leftarrow (0.0, -(\mu \times \beta \times I_0 \times \omega)/C_p)$
        $V[index] \leftarrow (k_s^2 - k_f^2, -\epsilon)$
      else
        $S[index] \leftarrow (0.0, 0.0)$
        $V[index] \leftarrow (0.0, -\epsilon)$
  // Rest of the code

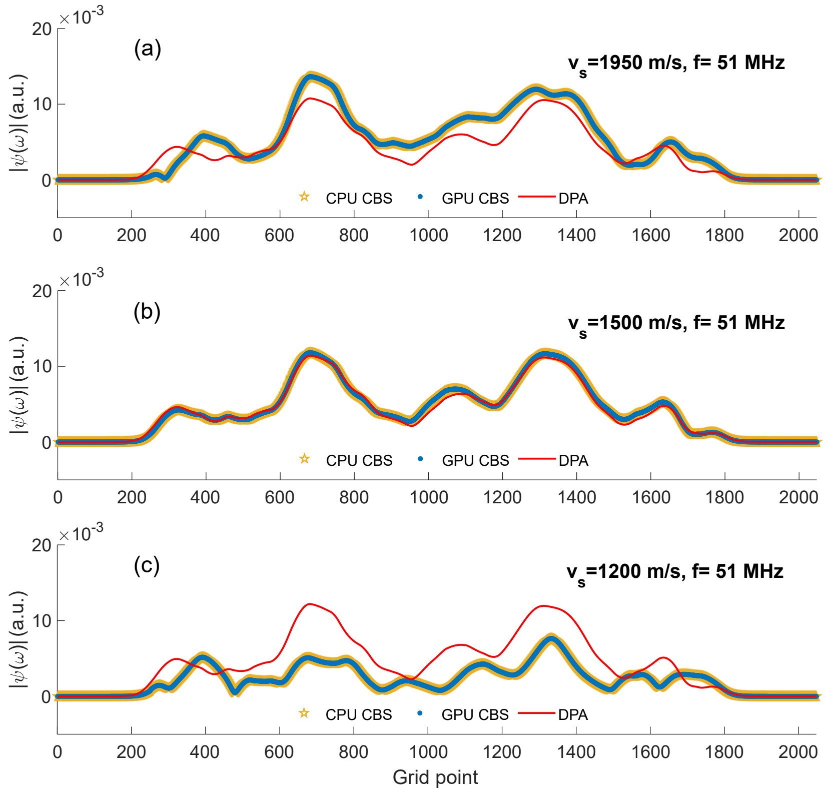

Figure S1: Plots of the amplitude of PA pressure computed at 51 MHz developed by a tissue realization along the center line [see Fig. 4(a)] at different sound-speed contrast conditions; v_s = 1950, 1500, 1200 m/s for (a), (b), (c), respectively and v_f = 1500 m/s.

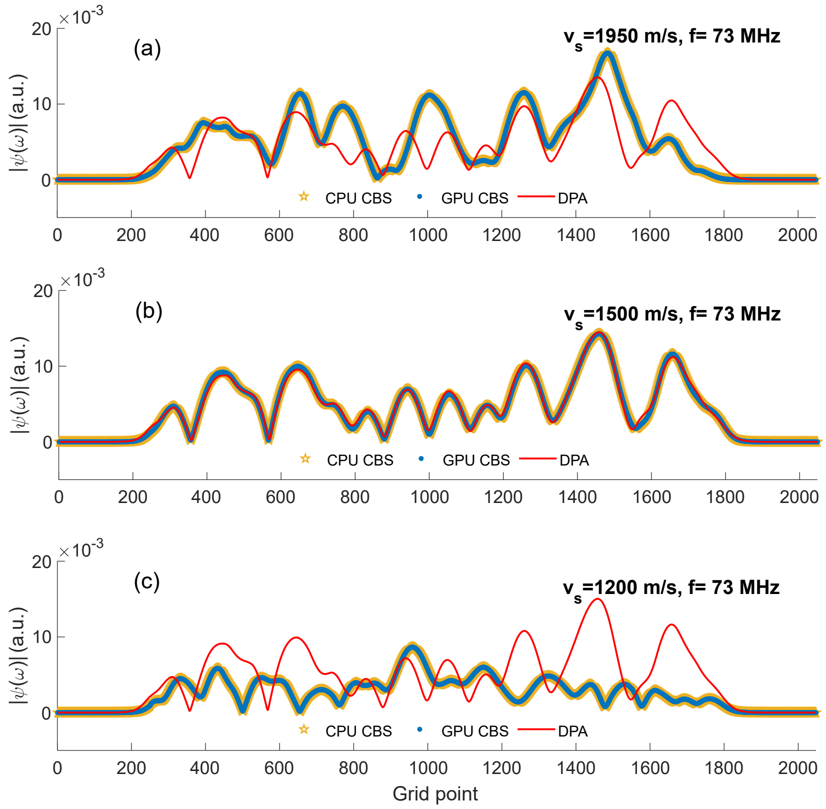

Figure S2: Same as Fig. S1 but for 73 MHz.

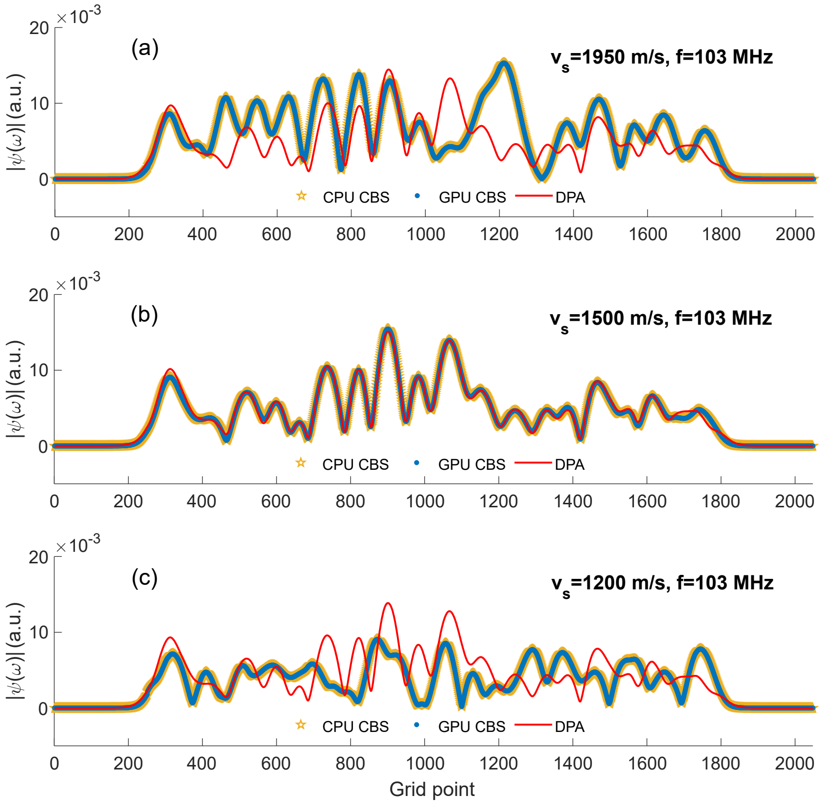

Figure S3: Same as Fig. S1 but for 103 MHz.

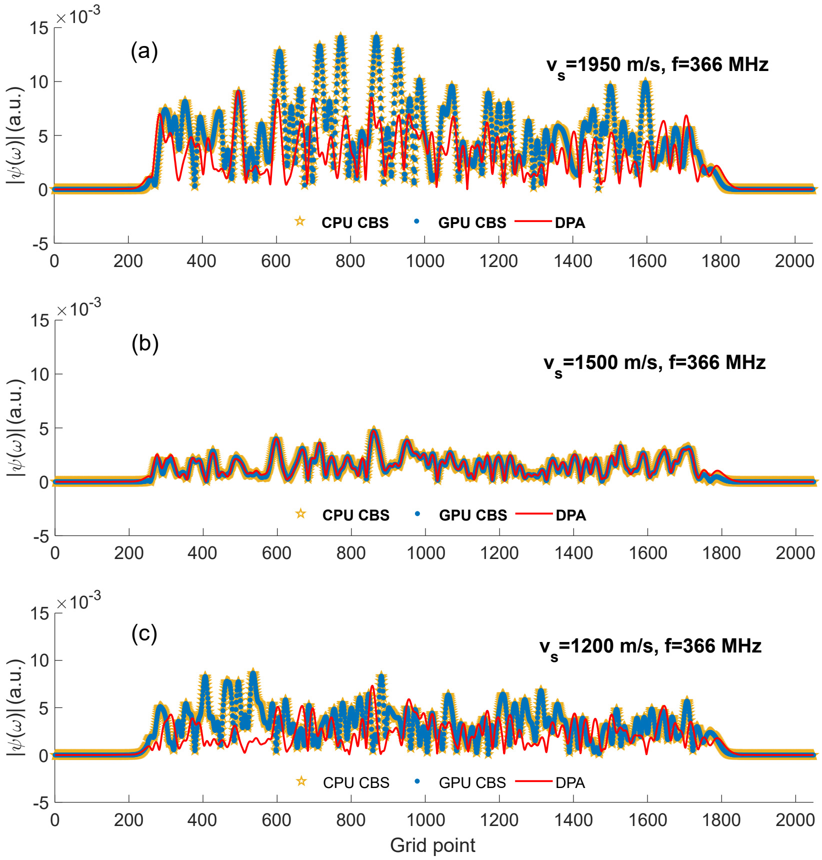

Figure S4: Same as Fig. S1 but for 366 MHz.

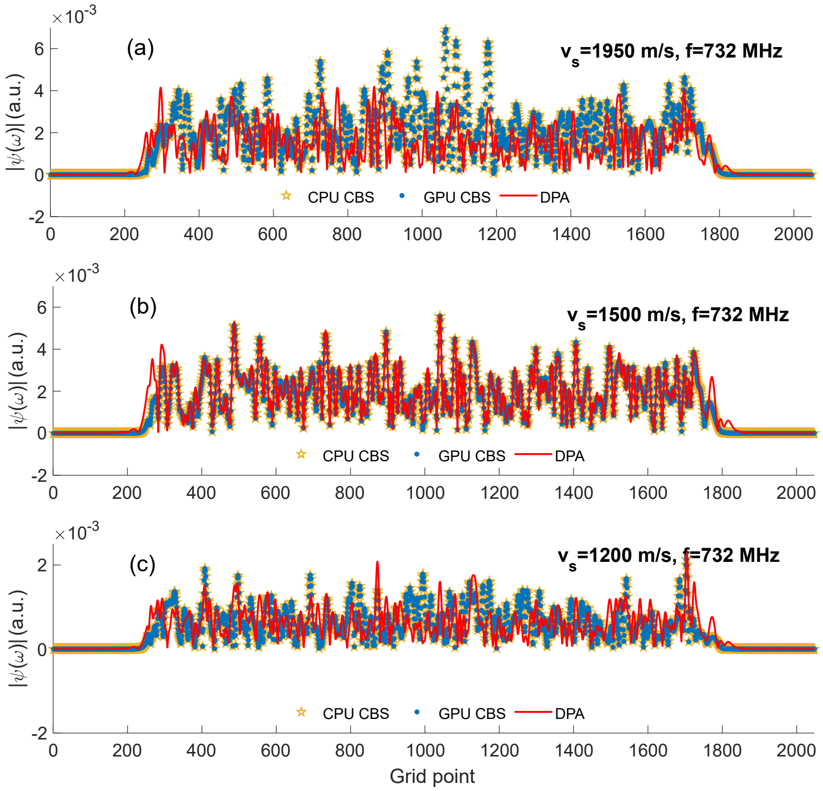

Figure S5: Same as Fig. S1 but for 732 MHz.
